# Association of telomere shortening in myocardium with heart weight gain and cause of death

**DOI:** 10.1038/srep02401

**Published:** 2013-08-09

**Authors:** Masanori Terai, Naotaka Izumiyama-Shimomura, Junko Aida, Naoshi Ishikawa, Motoji Sawabe, Tomio Arai, Mutsunori Fujiwara, Akio Ishii, Ken-ichi Nakamura, Kaiyo Takubo

**Affiliations:** 1 Research Team for Geriatric Pathology, Tokyo Metropolitan Institute of Gerontology, Tokyo 173-0015, Japan; 2 Department of Judotherapy, Faculty of Health Sciences, Tokyo Ariake University of Medical and Health Sciences, Tokyo 135-0063, Japan; 3 Department of Moleculo-genetic Sciences, Division of Biomedical Laboratory Sciences, Graduate School of Health Care Sciences, Tokyo Medical and Dental University, Tokyo, 113-8519, Japan; 4 Department of Pathology, Tokyo Metropolitan Geriatric Hospital, Tokyo 173-0015, Japan; 5 Department of Pathology, Japanese Red Cross Medical Center, Tokyo 150-8935, Japan

## Abstract

We attempted to clarify myocardial telomere dynamics using samples from 530 autopsied patients using Southern blot analysis. Overall regression analysis demonstrated yearly telomere reduction rate of 20 base pairs in the myocardium. There was a significant correlation between myocardial telomere and aging. Moreover, regression analyses of telomere and heart weight yielded a telomere reduction rate of 3 base pairs per gram, and a small but significant correlation between telomere reduction and heart weight was demonstrated. Hearts of autopsied patients who had died of heart disease were significantly heavier than those of patients who had died of cancer or other diseases, and heart disease was significantly more correlated with myocardial telomere shortening than cancer or other diseases. Here we show that telomeres in myocardial tissue become shortened with aging and heart disease, and that heart disease was associated with a gain of heart weight and telomere shortening in the myocardium.

Normal human cells exhibit a limited capacity for proliferation in culture^1^. This phenomenon is considered to be attributable to reduction of telomere length as an indicator of the number of divisions a cell has undergone. Telomeres protect chromosomes against degeneration, reconstruction, fusion and loss, as well as contributing to pairing of homologous chromosomes^2^,^3^. The end-to-end chromosome fusions observed in some tumors could play a role in genetic instability associated with tumorigenesis, and may be the result of telomere loss^2^. The human telomere is a simple repeating sequence of six bases, TTAGGG, located at the ends of chromosomes^4^.

We previously demonstrated that the yearly telomere reduction rate in 168 samples of myocardium from the anterior wall of the cardiac left ventricle was 13 base pairs, being similar to that in other human tissues and organs^5^. Our group has carried out a series of studies to measure telomere length in various human tissues, and reported telomere shortening with aging in the esophageal, gastric and colonic mucosae, the liver, and other sites^6^. Since telomere, as measured in human tissues by Southern blot analysis, demonstrates a very large standard deviation among individuals, attention has been concentrated on different tissues from many subjects^7^,^8^,^9^. Although available data on human tissue renewal times are limited, it is considered that very large differences exist among the various organs. Renewal of the gastrointestinal mucosal epithelium is very rapid, whereas that of hepatic and renal tissue is very slow. However, the yearly telomere reduction rates in human tissues and organs have been reported to be similar, at about 30–60 base pairs^5^. Myocardial tissue is exceptional in that it is relatively static with respect to cell turnover^10^, but no previous studies have investigated its telomere length status, moreover, there have been no previous attempts to examine the relationship between telomere length and age or cause of death. Therefore, the issue of age-related telomere shortening during life in the context of cause of death remains unclear.

Accordingly, in the present study using Southern blotting, we measured telomere in samples of myocardial tissue from a large sample of 530 autopsy cases covering a wide age range, from neonates to centenarians. Particular attention was paid to yearly telomere reduction rates in this large number of myocardium samples. Moreover, we analyzed the relationship between myocardial telomere and heart weight for major causes of death among the individuals examined.

## Results

**Figure 1:**
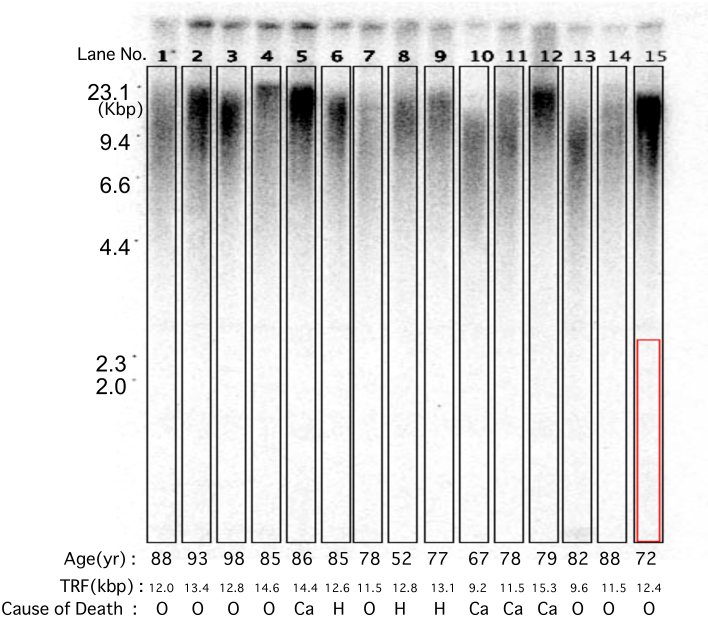
Image of a representative gel used for measurement of telomere length employing 15 samples. Genofield gel electrophoresis of genomic DNA from 15 samples used for assessment of DNA degradation. Molecular sizes (kbp) are indicated on the left. Age (yr), TRF (kbp) and cause of death (Ca; Cancer death, H; Heart disease death, O; Other causes of death) are listed at the bottom. The labeled boxes (black squares) were added by the authors using the Telomeric software package version 1.2, and the 15 lanes were analyzed to yield representative data for all TRF analyses (Southern blotting and quantification) that would allow evaluation of data quality. The labeled box (red square) in lane no.15 shows the location of an area suitable for background estimation, as it lies outside the DNA. For the constant field gels we analyzed, no smear effect was evident, and therefore we typically ran only a single marker lane.

**Figure 2:**
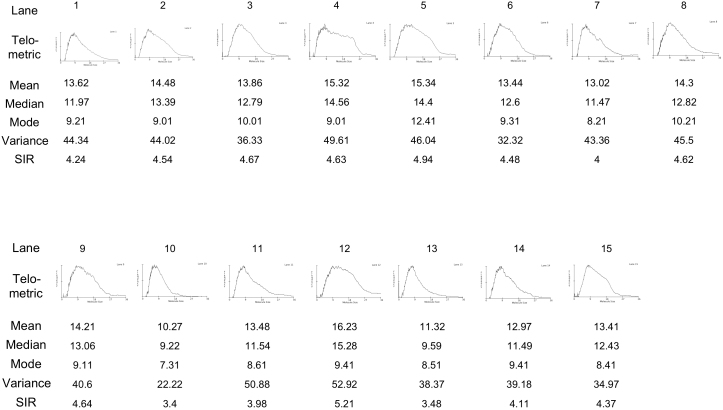
Plots of relative copy number versus telomere length for the 15 lanes outlined in Figure 1, and representative data obtained by statistical analysis using Telomeric version 1.2 based on the information in Figure 1. These plots were obtained by application of statistical analysis to raw data derived using Telomeric version 1.2 (15 samples) from the gel image shown in Figure 1. Since the distributions of telomere length and relative copy number are linear, the statistics generated were useful for determining the telomere length distribution to assess the sizes and distribution of TRF. A plot of relative copy number versus molecular weight is shown, which provides a realistic picture of the actual distribution of telomeric lengths. In addition, the distribution permits statistical information to be generated, including the mean, median, and mode of the molecular weight as well as the variance and the semi-interquartile range (SIR). For this gel with its asymmetric and skewed distribution, the significant values for determining the average telomere length and heterogeneity were the median and the SIR. The data are based on the results obtained by statistical analysis using Telomeric version 1.2 employing Figure 1. We adopted the median value of the TRF as being representative of telomere length.

Table 1 shows the data for age and cause of death among the 530 autopsied subjects. Five hundred and four of the subjects were aged 60 years or older. Southern blot analysis did not demonstrate bands, but rather smears of telomeric DNA. Supplementary information (A) shows a representative 1% agarose gel electrophoretogram stained with ethidium bromide for measurement of telomere length showing genomic DNA digested by *HinfI* (+) and non-digested genomic DNA (−) from 8 samples employed for verifying the process of digestion, including molecular size markers. Supplementary information (B) shows a representative 1% agarose gel electrophoretogram stained with ethidium bromide used for verifying the quality of genomic DNA for measurement of telomere length employing 19 samples, including molecular size markers. To explain how we analyzed terminal restriction fragment (TRF) using Southern blotting and quantification, representative results obtained using statistical analysis for the 530 autopsied subjects are shown as Figure 1 and Figure 2. Raw data for all 530 autopsied subjects are listed in Supplementary information (C) (age, sex, TRF and cause of death).

**Figure 3:**
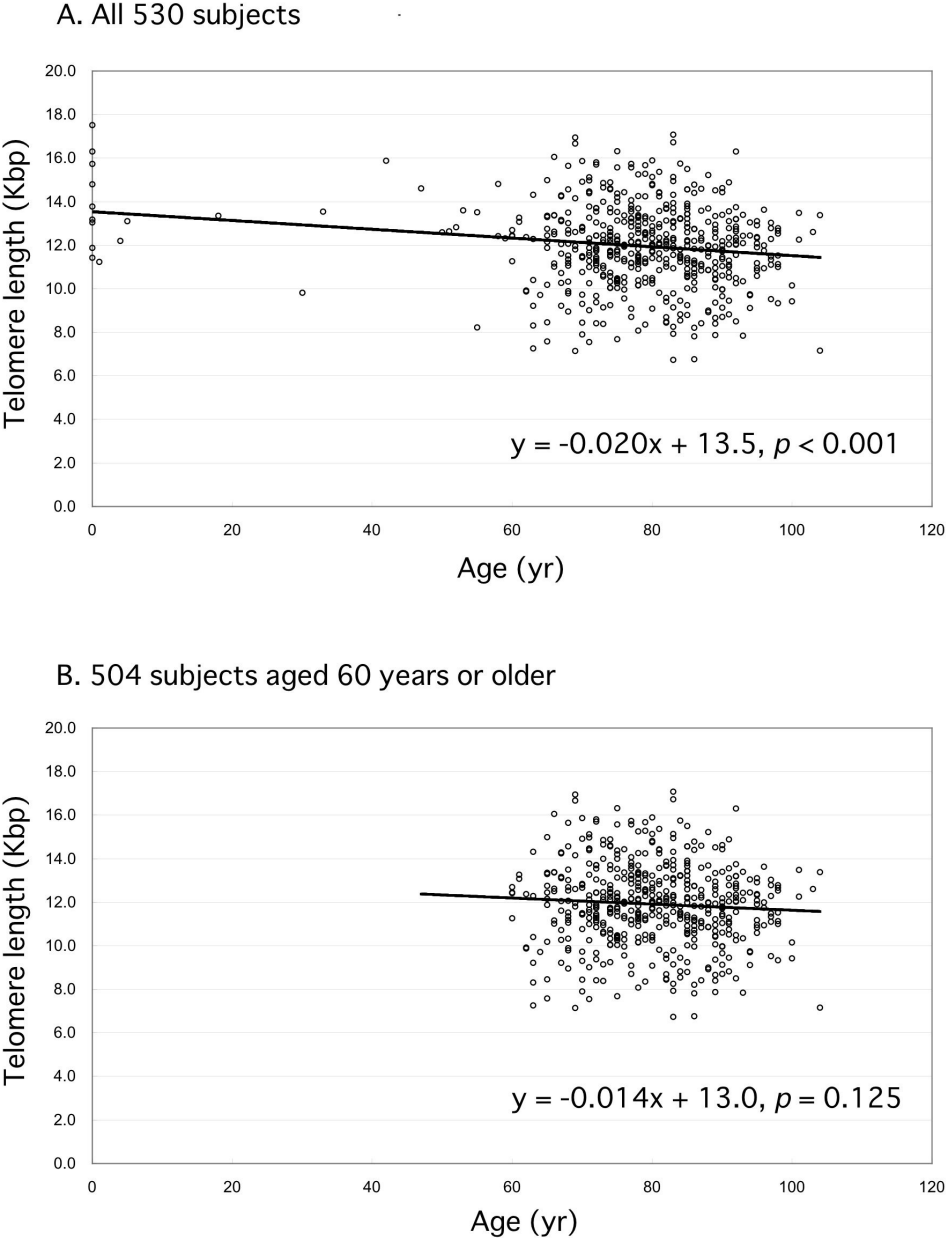
Scatter plots derived from regression analysis of the relationship between myocardial telomere length reduction and aging for samples taken from all 530 individuals in the present cohort (Fig.3 A) and the 504 patients aged 60 years or older (Fig.3 B). Regression analyses allowed the rates of myocardial telomere length reduction to be calculated as 20 (*p* < 0.001) and 14 (*p* = 0.125) base pairs per year in the 530 individuals overall (Fig.3 A) and the 504 patients aged 60 years or older (Fig.3 B), respectively. There was a significant reduction of myocardial telomere length with aging.

Figure 3 shows the results of regression analysis of the relationship between the annual telomere length reduction rate in the myocardium and aging, as scatter plots for samples taken from all 530 subjects (Fig. 3A), and for the 504 subjects who were aged 60 years or older (Fig. 3B). This allowed us to calculate the myocardial telomere length reduction rates as 20 (*p* < 0.001) and 14 (*p* = 0.125) base pairs per year for these two sets of subjects, respectively. The data indicated a significant age-related telomere length reduction in the myocardium for the 530 subjects overall, whereas the significance was only marginal for the 504 subjects aged 60 years or older. The data in Figure 3 include myocardial telomere lengths for 9 newborns (average 14.2 kbp, standard deviation 2.1 kbp) and 7 centenarians (average 11.2 kbp, standard deviation 2.4 kbp).

**Figure 4:**
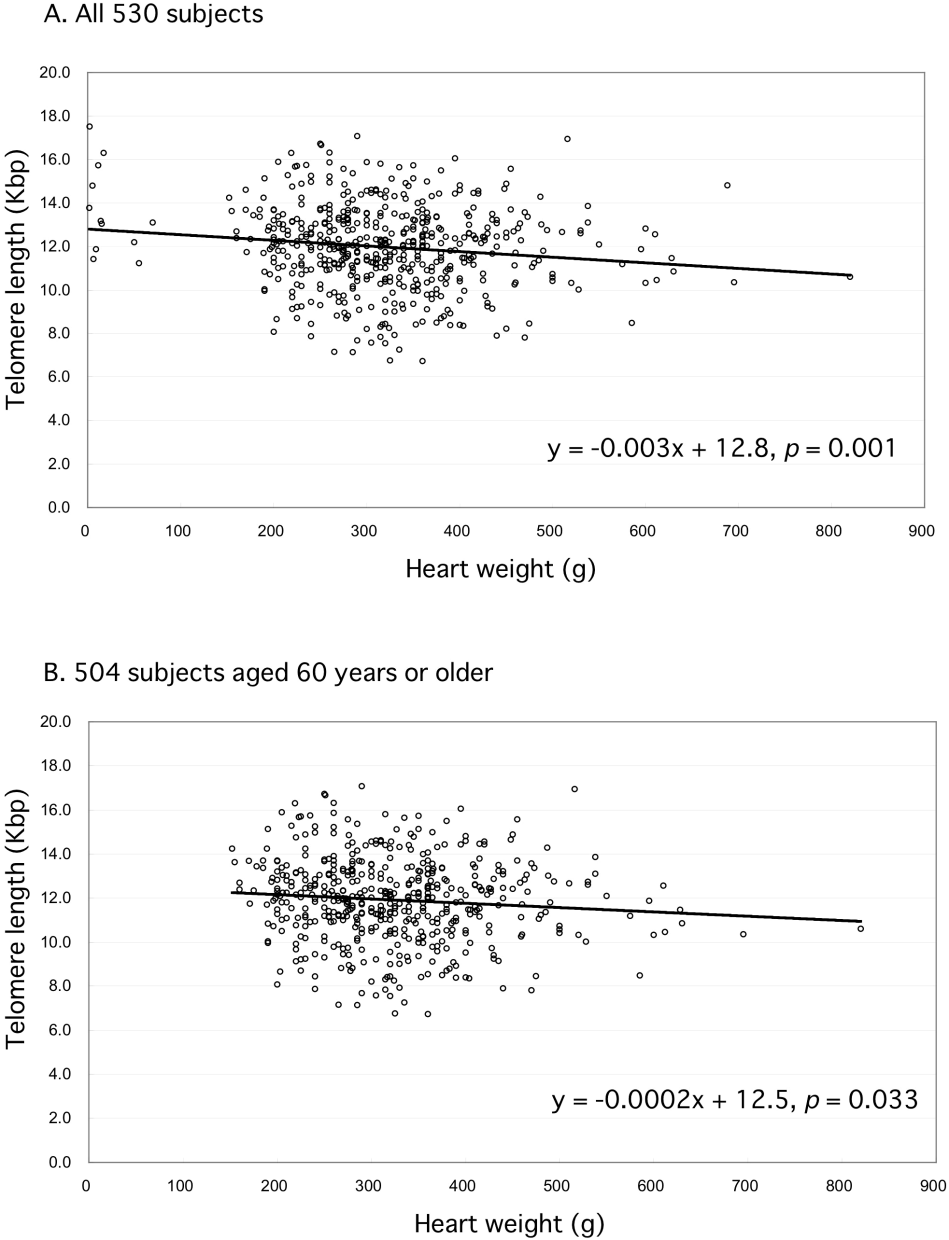
Scatter plots derived from regression analysis of the relationship between telomere length and heart weight for samples taken from all 530 individuals in the present cohort (Fig. 4A) and the 504 patients aged 60 years or older (Fig.4 B). Regression analyses allowed the myocardial telomere length reduction to be calculated as 3 (*p* = 0.001) and 0.2 (*p* = 0.033) base pairs per unit heart weight (gram) in the 530 subjects as a whole (Fig. 4A) and the 504 patients aged 60 years or older (Fig.4 B). The relationship between telomere length reduction and heart weight was small but significant.

Figure 4 shows the results of regression analysis of the relationship between the annual change in telomere length and heart weight (1.8 g ~ 820.0 g), as scatter plots for samples from all 530 subjects (Fig. 4A), and for the 504 who were aged 60 years or older (Fig. 4B). This allowed us to calculate the rates of myocardial telomere length reduction as 3 (*p* = 0.001) and 0.2 (*p* = 0.033) base pairs per unit heart weight (gram) for these two sets of subjects, respectively. The data indicated a small but significant correlation between myocardial telomere length and heart weight.

**Figure 5:**
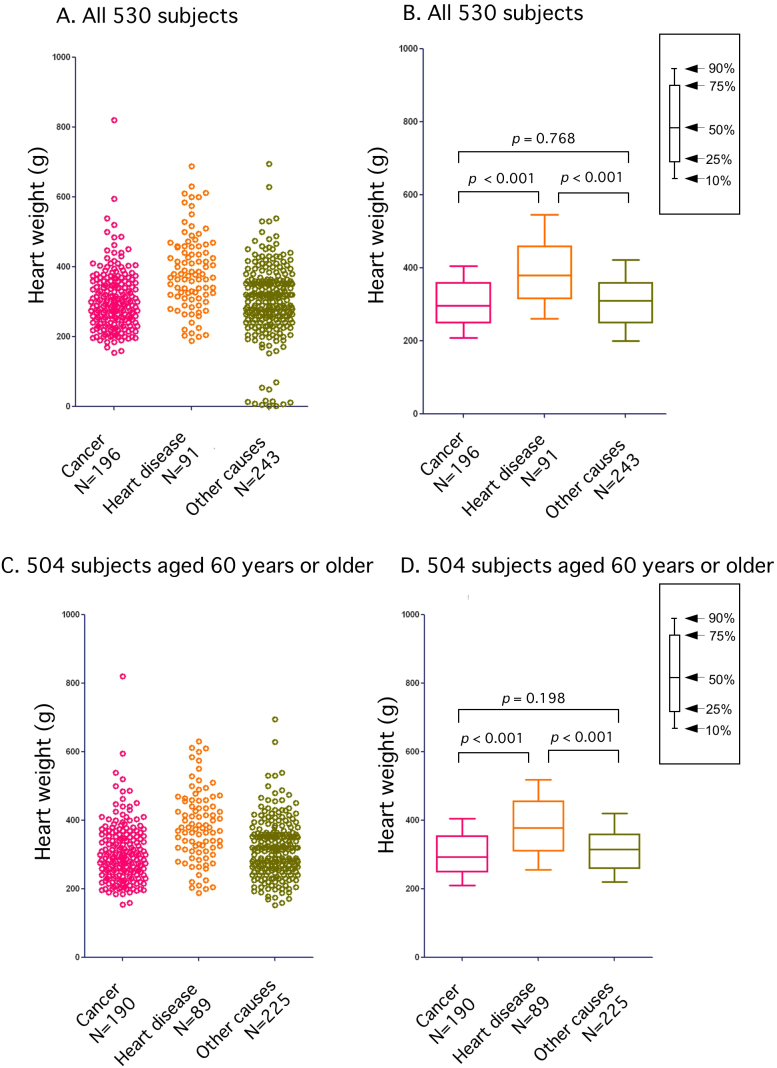
Heart weights for 530 of the study subjects (Fig.5 A and B- scatter plot and box plots) and the 504 patients aged 60 years or older (Fig.5 C and D- scatter plot and box plots), in relation to cause of death. Heart weight was related to cause of death; heart weight was significantly greater in subjects who had died of heart disease than in subjects who had died of cancer (Fig.5 B and D- box plots; *p* < 0.001) or other diseases (Fig.5 B and D- box plots; *p* < 0.001).

Figure 5 shows the relationship between cause of death and heart weight for all 530 subjects (Fig. 5A and B- scatter plot and box plots) and also the 504 patients aged 60 years or older (Fig. 5C and D- scatter plot and box plots). There was a strongly significant relationship between heart weight and cause of death; heart weight gain in individuals who had died of heart disease was more significant than in those who had died of cancer (*p* < 0.001) or other diseases (*p* < 0.001).

**Figure 6:**
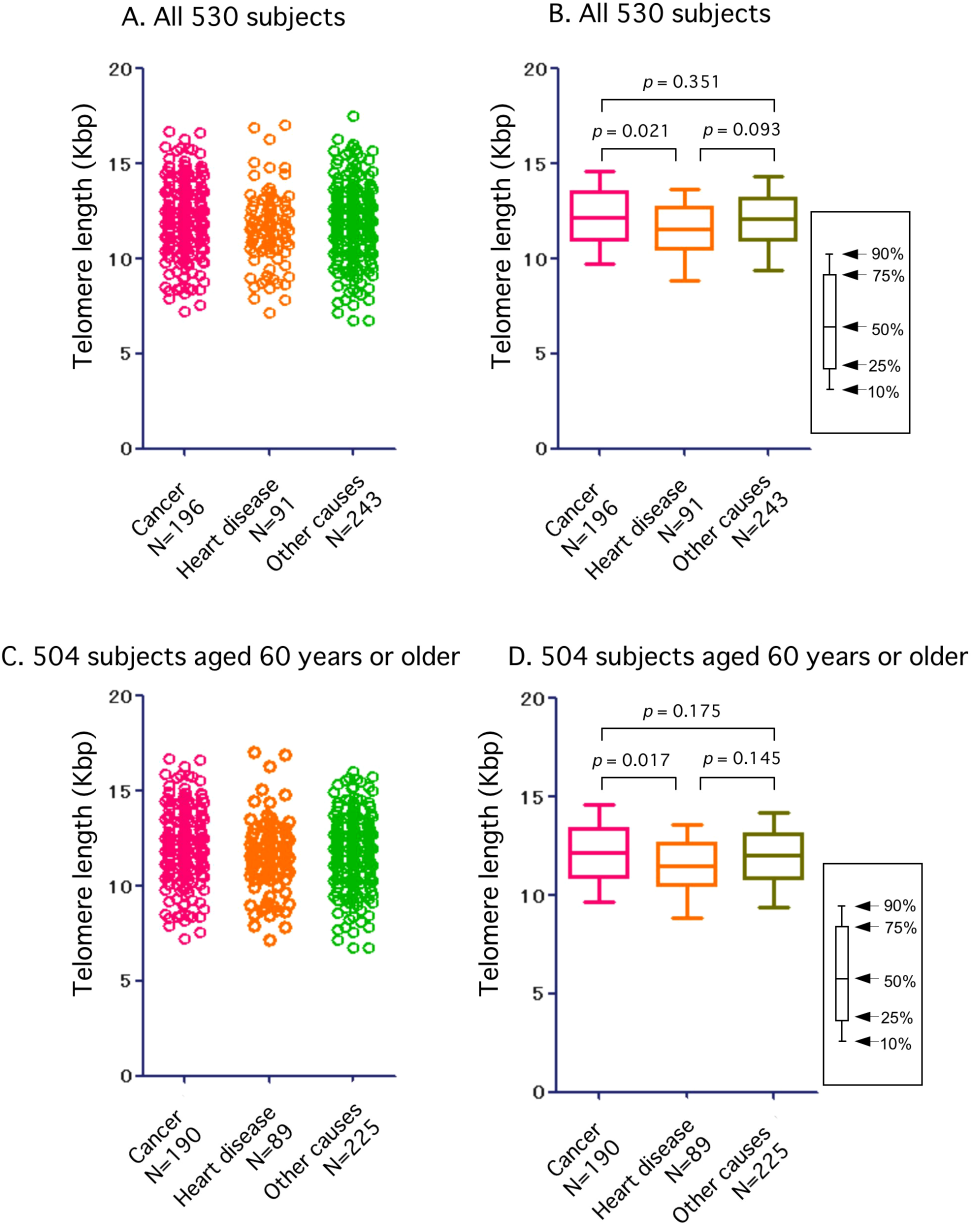
Myocardial telomere length for the 530 study subjects overall (Fig.6 A and B- scatter plot and box plots) and the 504 patients aged 60 years or older (Fig.6 C and D- scatter plot and box plots), in relation to cause of death. There was a significant correlation between myocardial telomere length and cause of death; myocardial telomeres in subjects who had died of heart disease were significantly shorter that in those who had died of cancer (Fig. 6B; *p* = 0.021, Fig.6 D; *p* = 0.017). However, myocardial telomeres in subjects who had died of heart disease were not significantly shorter than in those who had died of other diseases (Fig.6 B; 530 subjects; *p* = 0.093, Fig.6 D; 504 subjects; *p* = 0.145).

Figure 6 shows the relationship between myocardial telomere length and cause of death for all 530 subjects (Fig. 6A and B- scatter plot and box plots) and also the 504 patients aged 60 years or older (Fig. 6C and D- scatter plot and box plots). There was a significant correlation between myocardial telomere length and cause of death; myocardial telomeres in subjects who had died of heart disease were significantly shorter than in those who had died of cancer (Fig. 6B; 530 subjects; *p* = 0.021, Fig. 6D; 504 subjects; *p* = 0.017). However, myocardial telomeres in subjects who had died of heart disease were not significantly shorter than in those who had died of other diseases (Fig. 6B; 530 subjects; *p* = 0.093, Fig. 6D; 504 subjects; *p* = 0.145).

## Discussion

In previous studies, we have investigated the rates of telomere length reduction in 15 types of human cells, tissues and organs^5^. We have already investigated the association of telomere with aging using autopsy samples from the human pancreas^11^, cerebral gray and white matter^12^ and pituitary gland^13^. In 168 samples of myocardium from the anterior wall of the cardiac left ventricle, we previously demonstrated that the yearly rate of reduction of telomere length was 13 base pairs, although no significant telomere regression was observed in the myocardium^5^.

In the present study, therefore, we used Southern blotting to measure telomeres in a larger cohort of samples of quiescent myocardium from 530 autopsied individuals with a wide age range, from neonates to centenarians. Using regression analysis, we were able to demonstrate that the telomere reduction rate in the myocardium was 20 base pairs per year, and that there was a significant correlation between myocardial telomere and age.

Telomere reduction in the myocardium would be expected because fibroblasts and endothelial cells can undergo mitosis; but clearly this would not exert a major influence at the tissue level^5^. Telomeres in various tissues are significantly correlated within individuals; however, telomere in the myocardium is highly conserved, and the telomere reduction rate in any given individual was smaller than in other organs and tissues^5^.

In our study cohort, we found that telomeres in the myocardium became shorter with aging and heart disease. Although the significance of the correlation between myocardial telomere length and heart weight was small, it was more significant in subjects who had died of heart disease than in those who had died of cancer. We found that heart disease was associated with heart weight gain and telomere shortening in the myocardium.

Although from the 1920s it had been assumed that myocardial tissue is terminally differentiated and unable to undergo mitosis, recent years have seen a change in this viewpoint due to the discovery of myocardial cell division in the adult heart, and a net loss of total myocardium can be observed during physiological aging^14^,^15^,^16^. However, it had been thought that the increase in heart weight was due to hypertrophy without associated hyperplasia, since myocardium consists of terminally differentiated cells. Cardiac stem cells (CSCs) and cardiac progenitor cells (CPCs) are currently a center of interest^17^, and it is now clear that CSCs and CPCs develop from specific lineages and can differentiate in response to myocardial injury^18^,^19^,^20^. Although it has been thought that telomere dysfunction would lead to tissue atrophy, our present findings shed light on the association of myocardial differentiation and/or hypertrophy due to heart disease from the viewpoint of telomere length.

Although cardiac hypertrophy contributes to a gain in heart weight, there has been no evidence that this involves an increase in the number of cardiac cells. In the present study, however, we found that heart disease was associated with myocardial telomere shortening, thus supporting the possibility that that myocardium might regenerate and proliferate within the human lifespan as a result of heart disease or injury. However, any such regeneration might likely be limited to very slow myocardial replacement and growth. However, various attempts to regenerate the injured heart using cardiac stem cells, cellular reprogramming and tissue engineering are currently in progress^21^,^22^,^23^,^24^, and our present results could lead to a better understanding of how to treat or prevent heart failure in the future.

## Methods

The study protocol was approved by the Tokyo Metropolitan Institute of Gerontology Ethics Committee. Family members of all autopsied subjects gave written consent for educational and scientific use, including DNA analysis, of the subjects' organs. All experiments involving the handling of human tissues were performed in line with tenets of the Declaration of Helsinki.

After death, all the subjects were kept refrigerated at 4°C until autopsy. Tissues adjacent to all the sampled areas were examined histologically by specialists in anatomical and surgical pathology (M.S., T.A., M.F., and K.T.), and any tissues showing marked accumulation of inflammatory cells and/or autolysis were not included. Samples showing myocarditis were also avoided. Data on age and cause of death for the 530 subjects, who underwent autopsy at the Tokyo Metropolitan Geriatric Hospital and the Japanese Red Cross Medical Center, are shown in Table 1. Undegraded DNA samples were obtained from myocardium of the anterior wall of the cardiac left ventricle in all cases. One hundred sixty-eight of the 530 samples used in this study were from the series examined in our previous study^5^. After the tissue samples had been obtained in the autopsy room, they were frozen in liquid nitrogen and stored at −80°C until use.

DNA extraction and Southern blotting was performed by A.I. and K.N., who were blinded to data about age, cause of death and heart weight of the 530 autopsied subjects. Genomic DNA was prepared from each sample by treatment with proteinase K and sodium dodecyl sulphate (SDS), followed by repeated phenol-chloroform extraction. In preliminary experiments with genomic DNA from all samples, pulse-field gel electrophoresis was performed using 1.0% agarose gels and the Genofield system (ATTO, Tokyo, Japan), a biased sinusoidal field gel electrophoresis system that is able to examine autolytic changes after death. Only DNA more than 100 kilobase pairs (kbp) in length was assayed in this study. Aliquots of 5 μg were digested with the restriction enzyme *HinfI* (Boehringer Mannheim Biochemica, Germany); complete cleavage was confirmed by electrophoresis of the DNA digests on 0.8% agarose gels. Fractionated DNA fragments were transferred to nylon membranes (Hybond-N +, Amersham, UK) by an alkaline transfer technique using capillary blotting, followed by hybridization for 12 h at 50°C in an appropriate solution [6 × SSPE (1×; 0.15 M NaCl, 10 mM sodium phosphate, 1 mM EDTA, pH 7.4), 1% SDS] with a (TTAGGG)_4_ probe labeled with [*γ*-^32^P]ATP (Amersham) at the 5′ end with T4 polynucleotide kinase (Toyobo, Japan). Membranes were washed in 2 × SSC (NaCl 17.55 g/l, sodium citrate 8.82 g/l) at room temperature and then in 6 × SSC, 0.1% SDS, at 50°C for 15 min while being shaken. Subsequently, they were dried with filter paper and then exposed to Fuji Imaging Plates (Fuji Photo Film Co. Ltd., Japan) for 3 h at room temperature. Analysis was conducted with a BAS-2500 Mac image analyzer (Fuji Photo Film), employing the programs Image Reader (version 1.1, Fuji Photo Film) and Mac Bas (version 2.4, Fuji Photo Film). In this study, we used Telomeric software version 1.2 (Fox Case Cancer Center, USA) to assess the sizes and distribution TRF^25^. We adapted the median value of the TRF as a representative of telomere length, because the TRF values did not show a Gaussian distribution, and TRF was recorded for simplicity as telomere^5^,^26^.

Differences in mean values were assessed for significance by Student's *t* test and correlations by Fisher's test, using the methods reported in many previous studies of annual telomere reduction rates in myocardium^5^.

## Author Contributions

M.T. and K.T.: Conception and design, Data analysis and interpretation, Manuscript writing, Final approval of manuscript. N.I.-S., J.A., N.I., A.I. and K.N.: Collection and/or assembly of data, Data analysis and interpretation. M.S., T.A., M.F. and K.T.: Provision of study material or patients, Histological examination.

## Supplementary Material

Supplementary Information Supplementary information

## Figures and Tables

**Table 1:** Data for all 530 autopsied subjects (age and cause of death)

	Cause of death			
Subjects age	Cancer	Heart disease	Other causes	All causes
0–9 (yr)	0	0	12	12
10–19 (yr)	0	0	1	1
20–29 (yr)	0	0	0	0
30–39 (yr)	2	0	0	2
40–49 (yr)	1	0	1	2
50–59 (yr)	3	2	4	9
60–69 (yr)	29	11	24	64
70–79 (yr)	74	27	80	181
80–89 (yr)	67	29	73	169
90–99 (yr)	19	21	43	83
100–104 (yr)	1	1	5	7
Number of patients	196	91	243	530

## Notes

**Funding:** This work was supported by JSPS KAKENHI: 21390109 (K.T.) and 16590300 (K.N.).

## References

[b1] Hayflick L. The limited in vitro lifetime of human diploid cell strains. Exp. Cell Res. 37, 614–636 (1965). 14315085 10.1016/0014-4827(65)90211-9

[b2] Blackburn E. H. Structure and function of telomeres. Nature 350, 569–573 (1991). 1708110 10.1038/350569a0

[b3] Levy M. Z., Allsopp R. C., Futcher A. B., Greider C. W. & Harley C. B. Telomere end-replication problem and cell aging. J. Mol. Biol. 225, 951–960 (1992). 1613801 10.1016/0022-2836(92)90096-3

[b4] Moyzis R. K. *et al.* A highly conserved repetitive DNA sequence, (TTAGGG)n, present at the telomeres of human chromosomes. Proc. Natl Acad. Sci. USA 85, 6622–6626 (1988). 3413114 10.1073/pnas.85.18.6622 PMC282029

[b5] Takubo K. *et al.* Telomere lengths are characteristic in each human individual. Exp. Gerontol. 37, 523–531 (2002). 11830355 10.1016/s0531-5565(01)00218-2

[b6] Takubo K. *et al.* Changes of telomere length with aging. Geriatr. Gerontol. Int. 10, S197–206 (2010). 20590834 10.1111/j.1447-0594.2010.00605.x

[b7] Takubo K. *et al.* Telomere shortening with aging in human esophageal mucosa. Age 22, 95–99 (1999). 23604406 10.1007/s11357-999-0011-6 PMC3455805

[b8] Takubo K. *et al.* Telomere shortening with aging in human liver. J. Gerontol. A. Biol. Sci. Med. Sci. 55, B533–536 (2000). 11078086 10.1093/gerona/55.11.b533

[b9] Nakamura K. *et al.* Correlation of telomere lengths in normal and cancers tissue in the large bowel. Cancer Lett. 158, 179–184 (2000). 10960768 10.1016/s0304-3835(00)00521-8

[b10] Cameron I. L. Cell renewal in the organs and tissues of the nongrowing adult mouse. Tex. Rep. Biol. Med. 28, 203–248 (1970). 5521879

[b11] Ishii A. *et al.* Telomere shortening with aging in the human pancreas. Exp. Gerontol. 41, 882–886 (2006). 16860503 10.1016/j.exger.2006.06.036

[b12] Nakamura K. *et al.* Telomeric DNA length in cerebral gray and white matter is associated with longevity in individuals aged 70 years or older. Exp. Gerontol. 42, 944–950 (2007). 17606349 10.1016/j.exger.2007.05.003

[b13] Ishikawa N. *et al.* Telomere length dynamics in the human pituitary gland: robust preservation throughout adult life to centenarian age. Age (Dordr.) 34, 795–804 (2012). 21735056 10.1007/s11357-011-9280-y PMC3682069

[b14] Anversa P., Leri A. & Kajstura J. Cardiac regeneration. J. Am. Coll. Cardiol. 47, 1769–1776 (2006). 16682300 10.1016/j.jacc.2006.02.003

[b15] Chimenti C. *et al.* Senescence and death of primitive cells and myocytes lead to premature cardiac aging and heart failure. Circ. Res. 93, 604–613 (2003). 12958145 10.1161/01.RES.0000093985.76901.AF

[b16] Anversa P. *et al.* Myocyte cell loss and myocyte cellular hyperplasia in the hypertrophied aging rat heart. Circ. Res. 67, 871–885 (1990). 2145091 10.1161/01.res.67.4.871

[b17] Laflamme M. A. & Murry C. E. Heart regeneration. Nature 473, 326–335 (2011). 21593865 10.1038/nature10147 PMC4091722

[b18] Murry C. E. *et al.* Haematopoietic stem cells do not transdifferentiate into cardiac myocytes in myocardial infarcts. Nature 428, 664–668 (2004). 15034593 10.1038/nature02446

[b19] Yang L. *et al.* Human cardiovascular progenitor cells develop from a KDR+ embryonic-stem-cell-derived population. Nature 453, 524–528 (2008). 18432194 10.1038/nature06894

[b20] Bu L. *et al.* Human ISL1 heart progenitors generate diverse multipotent cardiovascular cell lineages. Nature 460, 113–117 (2009). 19571884 10.1038/nature08191

[b21] Miki K. *et al.* Bioengineered myocardium derived from induced pluripotent stem cells improves cardiac function and attenuates cardiac remodeling following chronic myocardial infarction in rats. Stem Cells Transl. Med. 1, 430–437 (2012). 23197822 10.5966/sctm.2011-0038 PMC3659710

[b22] Oka T. *et al.* Mitochondrial DNA that escapes from autophagy causes inflammation and heart failure. Nature 485, 251–255 (2012). 22535248 10.1038/nature10992 PMC3378041

[b23] Zhu W. *et al.* IGFBP-4 is an inhibitor of canonical Wnt signalling required for cardiogenesis. Nature 454, 345–349 (2008). 18528331 10.1038/nature07027

[b24] Sano M. *et al.* p53-induced inhibition of Hif-1 causes cardiac dysfunction during pressure overload. Nature 446, 444–448 (2007). 17334357 10.1038/nature05602

[b25] Grant J. D. *et al.* Telometric: A tool providing simplified, reproducible measurements of telomeric DNA from constant field agarose gels. BioTechniques 31, 1314–1318 (2001). 11768660 10.2144/01316bc02

[b26] Nakamura K.-I. *et al.* Comparative analysis of telomere lengths and erosion with age in human epidermis and lingual epithelium. J. Invest. Dermatol. 119, 1014–1019 (2002). 12445186 10.1046/j.1523-1747.2002.19523.x

