# Supplementary material for: Association of telomere shortening in myocardium with heart weight gain and cause of death
Source: Sci Rep. 2013 Aug 9;3:2401. doi: 10.1038/srep02401 (PMC3738945; doi:10.1038/srep02401)
Supplement: Supplementary Information [file srep02401-s1.doc]

**Supplementary information**

Association of telomere shortening in myocardium with heart weight gain and cause of death

Masanori Terai,^1,2^,*Naotaka Izumiyama-Shimomura,^1^ Junko Aida,^1^ Naoshi Ishikawa,^1^ Motoji Sawabe,^3^ Tomio Arai,^4^ Mutsunori Fujiwara,^5^ Akio Ishii,^1^ Ken-ichi Nakamura,^1^ and Kaiyo Takubo^1^,*

^1^Research Team for Geriatric Pathology, Tokyo Metropolitan Institute of Gerontology, Tokyo 173-0015, Japan

^2^Department of Judotherapy, Faculty of Health Sciences, Tokyo Ariake University of Medical and Health Sciences, Tokyo 135-0063, Japan

^3^Department of Moleculo-genetic Sciences, Division of Biomedical Laboratory Sciences, Graduate School of Health Care Sciences, Tokyo Medical and Dental University, Tokyo, 113-8519, Japan

^4^Department of Pathology, Tokyo Metropolitan Geriatric Hospital, Tokyo 173-0015, Japan

^5^Department of Pathology, Japanese Red Cross Medical Center, Tokyo 150-8935, Japan

***Corresponding authors:**

Masanori Terai, DDS, PhD

Department of Judotherapy,

Faculty of Health Sciences,

Tokyo Ariake University of Medical and Health Sciences,

2-9-1 Ariake, Koto-ku, Tokyo 135-0063, Japan

TEL&FAX: +81-3-6703-7061

E-mail: terai@tau.ac.jp

and

Kaiyo Takubo, MD

Research Team for Geriatric Pathology,

Tokyo Metropolitan Institute of Gerontology,

Tokyo 173-0015, Japan

FAX: +81-3-3579-4776

E-mail: ttaggg@tmig.or.jp

**Supplementary information**

**Supplementary information (A). Image of a representative 1% agarose gel electrophoretogram stained with ethidium bromide for measurement of telomere length showing genomic DNA digested by *HinfI* (+) and non-digested genomic DNA (-) from 8 samples employed for verifying the process of digestion, including molecular size markers.**

A representative 1% agarose gel electrophoretogram stained with ethidium bromide showing genomic DNA digested by *HinfI* (+) and non-digested genomic DNA (-) from 8 samples for verifying the process of digestion. Molecular sizes (kbp) are indicated on the left (φX174 RF DNA *HaeIII* Digested, BioLabs, USA). ID number, *HinfI*-digested (+) or non-digested (-), age (yr), TRF (kbp) and cause of death (Ca; Cancer death, H; Heart disease death, O; Other causes of death) are listed at the bottom. The 8 samples are listed according to the ID numbers shown in Supplementary information (C).

**Supplementary information (B). Image of a representative 1% agarose gel electrophoretogram stained with ethidium bromide used for verifying the quality of genomic DNA for measurement of telomere length employing 19 samples, including molecular size markers.**

A representative 1% agarose gel electrophoretogram stained with ethidium bromide showing genomic DNA from 19 samples employed for assessment of DNA quality. Molecular sizes (kbp) are indicated on the left (MidRange PFG Marker I, BioLabs, USA). ID number, age (yr), TRF (kbp) and cause of death (Ca; Cancer death, H; Heart disease death, O; Other causes of death) are listed at the bottom. The 19 samples are listed according to the ID numbers (3 to 21) shown in Supplementary information (C).

**Supplementary information (C). Raw data for all 530 autopsied subjects (age, sex, TRF and cause of death).**

**Supplementary information (A)**

**Supplementary information (B)**

**Supplementary information (C)**

| ID (Number) | Age | Sex | TRF(Kbp)/ Cancer death | TRF(Kbp)/Heart disease death | TRF(Kbp)/Other causes of death |
| --- | --- | --- | --- | --- | --- |
| 1 | 104 | F |  | 7.16 |  |
| 2 | 101 | F |  |  | 12.25 |
| 3 | 100 | F |  |  | 10.15 |
| 4 | 98 | F |  |  | 12.67 |
| 5 | 86 | F | 11.84 |  |  |
| 6 | 80 | M | 10.49 |  |  |
| 7 | 84 | F |  |  | 13.24 |
| 8 | 76 | M |  | 10.53 |  |
| 9 | 89 | M | 12.34 |  |  |
| 10 | 88 | M | 10.87 |  |  |
| 11 | 97 | M |  |  | 11.11 |
| 12 | 74 | M |  |  | 12.42 |
| 13 | 80 | F | 11.96 |  |  |
| 14 | 71 | M |  | 9.01 |  |
| 15 | 78 | M |  |  | 10.15 |
| 16 | 60 | F | 12.45 |  |  |
| 17 | 74 | M |  | 10.57 |  |
| 18 | 89 | F | 11.22 |  |  |
| 19 | 95 | F |  |  | 11.18 |
| 20 | 95 | F |  |  | 10.84 |
| 21 | 66 | M | 11.02 |  |  |
| 22 | 94 | F |  | 9.11 |  |
| 23 | 90 | F |  | 11.37 |  |
| 24 | 55 | M |  |  | 8.22 |
| 25 | 47 | F | 14.61 |  |  |
| 26 | 69 | M | 12.61 |  |  |
| 27 | 65 | F |  |  | 11.87 |
| 28 | 67 | F | 10.72 |  |  |
| 29 | 68 | F | 11.35 |  |  |
| 30 | 62 | M | 9.92 |  |  |
| 31 | 68 | F | 13.01 |  |  |
| 32 | 70 | M |  | 8.45 |  |
| 33 | 83 | F | 11.86 |  |  |
| 34 | 65 | F |  |  | 13.3 |
| 35 | 95 | F |  | 11.9 |  |
| 36 | 90 | F |  |  | 11.81 |
| 37 | 90 | M | 10.61 |  |  |
| 38 | 90 | M | 11.71 |  |  |
| 39 | 92 | M |  |  | 11.56 |
| 40 | 92 | F |  |  | 10.49 |
| 41 | 91 | F |  |  | 10.52 |
| 42 | 90 | F |  |  | 12 |
| 43 | 95 | F |  |  | 12.37 |
| 44 | 92 | F |  | 12.03 |  |
| 45 | 91 | M |  |  | 11.11 |
| 46 | 74 | F | 9.25 |  |  |
| 47 | 90 | F | 9.13 |  |  |
| 48 | 68 | M |  | 11.19 |  |
| 49 | 80 | M | 12.05 |  |  |
| 50 | 79 | M | 8.36 |  |  |
| 51 | 75 | M | 10.04 |  |  |
| 52 | 84 | M | 11.23 |  |  |
| 53 | 75 | F | 10.26 |  |  |
| 54 | 82 | M |  | 8.49 |  |
| 55 | 72 | F | 8.41 |  |  |
| 56 | 82 | F | 13.71 |  |  |
| 57 | 77 | F |  | 8.7 |  |
| 58 | 74 | M | 10.74 |  |  |
| 59 | 72 | M | 11.17 |  |  |
| 60 | 65 | M | 12.45 |  |  |
| 61 | 79 | M |  | 13.32 |  |
| 62 | 84 | M |  |  | 13.22 |
| 63 | 84 | F |  |  | 8.55 |
| 64 | 86 | F | 8.21 |  |  |
| 65 | 71 | M |  |  | 7.55 |
| 66 | 83 | F | 7.93 |  |  |
| 67 | 78 | M | 12.09 |  |  |
| 68 | 83 | F |  |  | 6.73 |
| 69 | 86 | F |  |  | 6.76 |
| 70 | 87 | M | 8.42 |  |  |
| 71 | 88 | F |  | 8.93 |  |
| 72 | 76 | F |  |  | 9.55 |
| 73 | 85 | F |  |  | 13 |
| 74 | 89 | M | 12.94 |  |  |
| 75 | 89 | F |  | 12.6 |  |
| 76 | 90 | F |  |  | 9.97 |
| 77 | 90 | M |  |  | 9.98 |
| 78 | 63 | F |  |  | 10.4 |
| 79 | 81 | M |  | 13.57 |  |
| 80 | 89 | M |  | 8.67 |  |
| 81 | 89 | M |  |  | 7.87 |
| 82 | 63 | F |  |  | 9.22 |
| 83 | 89 | F | 10.06 |  |  |
| 84 | 83 | M |  |  | 13.11 |
| 85 | 97 | F | 11.48 |  |  |
| 86 | 94 | F | 9.75 |  |  |
| 87 | 81 | M | 14.41 |  |  |
| 88 | 77 | M |  |  | 13.07 |
| 89 | 69 | M |  |  | 7.15 |
| 90 | 93 | F |  |  | 7.84 |
| 91 | 96 | F |  | 11.74 |  |
| 92 | 84 | F |  |  | 8.82 |
| 93 | 92 | M |  |  | 10.25 |
| 94 | 83 | M |  |  | 13.93 |
| 95 | 92 | M |  |  | 12.74 |
| 96 | 89 | M |  |  | 12.74 |
| 97 | 74 | M | 10.33 |  |  |
| 98 | 84 | F |  |  | 11.8 |
| 99 | 65 | F |  |  | 8.46 |
| 100 | 97 | F |  |  | 9.52 |
| 101 | 75 | F |  | 10.35 |  |
| 102 | 71 | F |  |  | 11.28 |
| 103 | 77 | F | 14.07 |  |  |
| 104 | 80 | F |  | 12.66 |  |
| 105 | 84 | M |  |  | 11.47 |
| 106 | 73 | F | 14.65 |  |  |
| 107 | 92 | M |  |  | 12.22 |
| 108 | 64 | F |  |  | 9.71 |
| 109 | 78 | M |  |  | 12.15 |
| 110 | 92 | F | 8.5 |  |  |
| 111 | 72 | M | 11.88 |  |  |
| 112 | 68 | M | 8.96 |  |  |
| 113 | 63 | M | 7.26 |  |  |
| 114 | 63 | M |  |  | 12.29 |
| 115 | 73 | M | 14.86 |  |  |
| 116 | 79 | M |  |  | 13.02 |
| 117 | 97 | F |  |  | 11.33 |
| 118 | 94 | F |  |  | 9.7 |
| 119 | 89 | M |  |  | 10.68 |
| 120 | 76 | M |  | 11.99 |  |
| 121 | 90 | M |  | 8.71 |  |
| 122 | 72 | M |  |  | 11.73 |
| 123 | 76 | M | 11.93 |  |  |
| 124 | 92 | F | 13.75 |  |  |
| 125 | 65 | M |  |  | 10.18 |
| 126 | 78 | M | 11.13 |  |  |
| 127 | 79 | M |  |  | 10.28 |
| 128 | 91 | F |  | 11.29 |  |
| 129 | 91 | M |  |  | 11.02 |
| 130 | 101 | F | 13.48 |  |  |
| 131 | 76 | F | 13.37 |  |  |
| 132 | 91 | F |  |  | 9.45 |
| 133 | 93 | M |  | 12.09 |  |
| 134 | 94 | F |  | 13.04 |  |
| 135 | 70 | M |  | 10.77 |  |
| 136 | 72 | M |  |  | 9.38 |
| 137 | 76 | F |  | 11.37 |  |
| 138 | 78 | M |  |  | 13.69 |
| 139 | 69 | F | 14.16 |  |  |
| 140 | 104 | F |  |  | 15.5 |
| 141 | 85 | M |  |  | 14.43 |
| 142 | 76 | M |  |  | 13.5 |
| 143 | 68 | M |  |  | 13.54 |
| 144 | 77 | M | 15.76 |  |  |
| 145 | 82 | M | 12.05 |  |  |
| 146 | 78 | M |  |  | 13.07 |
| 147 | 85 | M |  |  | 13.91 |
| 148 | 74 | M | 12.26 |  |  |
| 149 | 68 | M |  |  | 15.64 |
| 150 | 86 | M | 14.79 |  |  |
| 151 | 74 | M |  | 11.72 |  |
| 152 | 72 | F |  |  | 10.86 |
| 153 | 74 | F |  |  | 12.61 |
| 154 | 95 | F |  |  | 12.08 |
| 155 | 94 | F |  | 12.44 |  |
| 156 | 76 | M |  |  | 13.07 |
| 157 | 91 | M | 14.88 |  |  |
| 158 | 63 | F | 8.31 |  |  |
| 159 | 89 | F |  | 10.96 |  |
| 160 | 83 | M | 13.71 |  |  |
| 161 | 77 | F |  |  | 15.57 |
| 162 | 63 | F | 14.32 |  |  |
| 163 | 81 | F |  |  | 14.24 |
| 164 | 66 | M | 11.16 |  |  |
| 165 | 77 | F | 12.45 |  |  |
| 166 | 73 | F |  |  | 14 |
| 167 | 82 | M | 12.15 |  |  |
| 168 | 78 | M | 12.57 |  |  |
| 169 | 79 | M | 11.38 |  |  |
| 170 | 85 | F | 13.66 |  |  |
| 171 | 82 | F |  |  | 13.67 |
| 172 | 77 | M | 11.19 |  |  |
| 173 | 80 | M |  | 12.57 |  |
| 174 | 61 | M |  |  | 13.07 |
| 175 | 81 | M |  |  | 12.03 |
| 176 | 61 | F | 13.26 |  |  |
| 177 | 78 | F |  |  | 12.73 |
| 178 | 85 | M | 11.98 |  |  |
| 179 | 80 | F | 11.41 |  |  |
| 180 | 68 | M | 9.79 |  |  |
| 181 | 68 | F | 12.7 |  |  |
| 182 | 95 | M | 13.1 |  |  |
| 183 | 85 | M | 14.57 |  |  |
| 184 | 80 | M | 13.22 |  |  |
| 185 | 90 | M |  | 11.05 |  |
| 186 | 97 | F | 12.79 |  |  |
| 187 | 90 | F | 13.4 |  |  |
| 188 | 79 | F |  |  | 12.29 |
| 189 | 80 | F |  | 11.69 |  |
| 190 | 71 | F |  |  | 12.13 |
| 191 | 83 | F | 12.24 |  |  |
| 192 | 74 | M | 11.75 |  |  |
| 193 | 85 | F |  |  | 13.44 |
| 194 | 74 | M | 14.89 |  |  |
| 195 | 75 | M |  |  | 10.36 |
| 196 | 85 | F | 10.53 |  |  |
| 197 | 69 | M | 11.81 |  |  |
| 198 | 89 | M |  | 10.46 |  |
| 199 | 83 | F | 12.24 |  |  |
| 200 | 88 | M |  |  | 11.64 |
| 201 | 77 | M | 11.62 |  |  |
| 202 | 71 | M | 14.46 |  |  |
| 203 | 92 | F |  |  | 12.35 |
| 204 | 67 | M |  |  | 10.27 |
| 205 | 78 | M |  |  | 10.38 |
| 206 | 77 | F |  |  | 13.23 |
| 207 | 90 | F | 12.18 |  |  |
| 208 | 77 | M | 10.99 |  |  |
| 209 | 85 | F |  |  | 11.31 |
| 210 | 72 | M | 11.28 |  |  |
| 211 | 78 | M |  |  | 11.23 |
| 212 | 71 | F | 14.97 |  |  |
| 213 | 67 | F | 11.61 |  |  |
| 214 | 91 | M |  |  | 12.34 |
| 215 | 70 | F |  |  | 12.8 |
| 216 | 66 | M | 12.5 |  |  |
| 217 | 87 | F |  |  | 11.13 |
| 218 | 81 | M | 11.63 |  |  |
| 219 | 80 | M | 12.32 |  |  |
| 220 | 79 | F | 12.6 |  |  |
| 221 | 86 | F |  | 12.2 |  |
| 222 | 79 | F | 10.5 |  |  |
| 223 | 82 | F |  | 11.16 |  |
| 224 | 86 | M | 10.58 |  |  |
| 225 | 98 | F |  |  | 11.13 |
| 226 | 91 | F |  |  | 12.04 |
| 227 | 72 | F |  |  | 13.05 |
| 228 | 81 | M |  | 12.09 |  |
| 229 | 85 | F |  |  | 13.42 |
| 230 | 85 | M | 13.22 |  |  |
| 231 | 83 | M |  |  | 14.95 |
| 232 | 82 | M |  |  | 12.5 |
| 233 | 82 | F |  |  | 8.69 |
| 234 | 76 | M |  |  | 9.96 |
| 235 | 83 | F |  |  | 8.25 |
| 236 | 93 | M |  | 10.35 |  |
| 237 | 78 | F |  |  | 14.26 |
| 238 | 91 | F |  | 13.56 |  |
| 239 | 75 | M | 10.45 |  |  |
| 240 | 89 | M |  |  | 9.36 |
| 241 | 74 | F |  |  | 13.51 |
| 242 | 75 | M |  |  | 14.38 |
| 243 | 68 | M |  | 12.06 |  |
| 244 | 70 | M |  |  | 11.44 |
| 245 | 75 | F |  |  | 10.42 |
| 246 | 87 | F |  | 10.84 |  |
| 247 | 84 | M |  | 9.45 |  |
| 248 | 71 | M |  | 11.54 |  |
| 249 | 75 | F | 13.99 |  |  |
| 250 | 83 | F |  |  | 10.94 |
| 251 | 77 | F | 12.16 |  |  |
| 252 | 98 | F |  | 11 |  |
| 253 | 90 | M |  |  | 11.78 |
| 254 | 76 | M |  |  | 12.03 |
| 255 | 80 | M | 12.87 |  |  |
| 256 | 78 | F |  |  | 13.38 |
| 257 | 74 | F |  | 13.7 |  |
| 258 | 72 | M | 11.36 |  |  |
| 259 | 87 | F |  |  | 13.59 |
| 260 | 75 | M |  |  | 13.53 |
| 261 | 89 | F |  |  | 14.52 |
| 262 | 80 | F | 11.95 |  |  |
| 263 | 85 | F |  |  | 13.79 |
| 264 | 79 | M |  |  | 11.18 |
| 265 | 78 | M |  |  | 10.17 |
| 266 | 74 | M | 10.04 |  |  |
| 267 | 80 | F | 10.24 |  |  |
| 268 | 78 | M |  |  | 11.3 |
| 269 | 74 | M |  |  | 12.12 |
| 270 | 93 | M | 12.43 |  |  |
| 271 | 74 | M |  |  | 10.05 |
| 272 | 89 | F | 11.94 |  |  |
| 273 | 79 | M |  |  | 11.25 |
| 274 | 78 | M |  |  | 11.41 |
| 275 | 85 | M | 10.7 |  |  |
| 276 | 73 | F |  |  | 8.38 |
| 277 | 83 | M | 13.5 |  |  |
| 278 | 84 | F |  |  | 13.2 |
| 279 | 97 | F |  | 13.03 |  |
| 280 | 72 | M |  |  | 13.71 |
| 281 | 85 | M | 12.17 |  |  |
| 282 | 78 | M |  |  | 12.89 |
| 283 | 89 | M |  |  | 13.34 |
| 284 | 76 | F |  | 11.27 |  |
| 285 | 69 | F |  |  | 11.97 |
| 286 | 83 | M |  |  | 13.04 |
| 287 | 87 | M | 14.59 |  |  |
| 288 | 70 | M | 12.84 |  |  |
| 289 | 74 | M | 14.61 |  |  |
| 290 | 90 | F |  | 14.44 |  |
| 291 | 82 | F |  |  | 11.01 |
| 292 | 76 | M |  |  | 10.28 |
| 293 | 75 | F | 15.57 |  |  |
| 294 | 97 | M |  |  | 12.29 |
| 295 | 103 | F |  |  | 12.61 |
| 296 | 85 | M | 10.59 |  |  |
| 297 | 85 | F |  | 10.02 |  |
| 298 | 79 | M |  |  | 12.55 |
| 299 | 84 | M |  |  | 11.4 |
| 300 | 92 | F |  |  | 12.64 |
| 301 | 84 | M | 11.54 |  |  |
| 302 | 86 | F | 12.76 |  |  |
| 303 | 60 | M |  | 12.7 |  |
| 304 | 86 | F |  |  | 11.19 |
| 305 | 77 | F | 12.15 |  |  |
| 306 | 74 | M |  | 12.79 |  |
| 307 | 81 | F |  |  | 14.43 |
| 308 | 92 | F |  | 13.2 |  |
| 309 | 78 | F |  |  | 8.08 |
| 310 | 72 | M | 11.8 |  |  |
| 311 | 79 | M |  |  | 14.24 |
| 312 | 80 | M | 14.76 |  |  |
| 313 | 92 | M |  |  | 13.19 |
| 314 | 70 | M |  |  | 12.67 |
| 315 | 77 | F |  |  | 15.74 |
| 316 | 74 | F | 14.56 |  |  |
| 317 | 81 | M | 12.34 |  |  |
| 318 | 75 | M |  |  | 13.13 |
| 319 | 98 | F | 12.54 |  |  |
| 320 | 83 | M | 14.32 |  |  |
| 321 | 67 | F |  | 13.11 |  |
| 322 | 83 | F |  |  | 11.55 |
| 323 | 86 | M |  |  | 12.74 |
| 324 | 74 | M |  |  | 11.55 |
| 325 | 78 | F |  |  | 15.25 |
| 326 | 83 | M | 12.33 |  |  |
| 327 | 73 | F |  |  | 12.24 |
| 328 | 74 | F | 12.46 |  |  |
| 329 | 87 | M | 12.56 |  |  |
| 330 | 71 | F | 13.78 |  |  |
| 331 | 70 | F | 15.86 |  |  |
| 332 | 78 | F |  |  | 13.27 |
| 333 | 77 | M |  |  | 13.82 |
| 334 | 78 | F | 10.91 |  |  |
| 335 | 74 | F |  | 11.67 |  |
| 336 | 86 | M |  |  | 10.72 |
| 337 | 55 | F | 13.52 |  |  |
| 338 | 75 | F |  | 12.2 |  |
| 339 | 65 | M |  |  | 12.15 |
| 340 | 82 | F |  |  | 12.23 |
| 341 | 78 | M |  |  | 11.83 |
| 342 | 74 | M | 11.49 |  |  |
| 343 | 95 | F |  |  | 10.59 |
| 344 | 87 | M | 12.31 |  |  |
| 345 | 81 | M | 12.83 |  |  |
| 346 | 73 | M |  |  | 10.94 |
| 347 | 75 | M | 10.47 |  |  |
| 348 | 96 | F |  |  | 10.94 |
| 349 | 85 | M | 12.23 |  |  |
| 350 | 82 | M | 13.67 |  |  |
| 351 | 66 | F |  |  | 12.62 |
| 352 | 81 | M | 10.86 |  |  |
| 353 | 73 | M |  | 11.06 |  |
| 354 | 95 | M | 10.96 |  |  |
| 355 | 82 | M |  |  | 11.37 |
| 356 | 62 | F |  |  | 12.37 |
| 357 | 91 | M |  |  | 12.79 |
| 358 | 90 | F |  |  | 14.62 |
| 359 | 98 | F |  |  | 11.53 |
| 360 | 71 | F | 11.87 |  |  |
| 361 | 75 | F |  |  | 11.36 |
| 362 | 68 | M |  | 10.33 |  |
| 363 | 75 | F |  |  | 12.07 |
| 364 | 67 | M | 14.29 |  |  |
| 365 | 92 | F | 16.31 |  |  |
| 366 | 72 | M | 15.81 |  |  |
| 367 | 80 | F | 15.9 |  |  |
| 368 | 83 | M | 16.73 |  |  |
| 369 | 75 | M |  | 16.32 |  |
| 370 | 72 | M |  |  | 15.7 |
| 371 | 69 | F | 16.67 |  |  |
| 372 | 88 | F |  |  | 15.22 |
| 373 | 85 | M |  |  | 11.18 |
| 374 | 83 | M |  |  | 9.79 |
| 375 | 83 | F |  | 17.08 |  |
| 376 | 89 | M | 10.42 |  |  |
| 377 | 88 | F |  |  | 9 |
| 378 | 84 | F | 15.75 |  |  |
| 379 | 72 | F | 13.55 |  |  |
| 380 | 75 | F | 12.42 |  |  |
| 381 | 91 | M |  | 8.83 |  |
| 382 | 79 | M | 15.68 |  |  |
| 383 | 70 | M | 14.91 |  |  |
| 384 | 75 | F | 14.2 |  |  |
| 385 | 72 | F |  |  | 13.7 |
| 386 | 95 | F |  |  | 12.92 |
| 387 | 77 | M | 12.85 |  |  |
| 388 | 65 | F |  |  | 14.99 |
| 389 | 92 | M |  |  | 12.86 |
| 390 | 77 | M | 13.67 |  |  |
| 391 | 81 | M |  |  | 15.37 |
| 392 | 69 | M |  | 16.94 |  |
| 393 | 96 | M | 12 |  |  |
| 394 | 80 | F |  | 14.81 |  |
| 395 | 96 | F | 13.63 |  |  |
| 396 | 88 | M |  |  | 13.08 |
| 397 | 68 | M |  |  | 14.56 |
| 398 | 77 | M |  |  | 13.23 |
| 399 | 82 | M | 13.86 |  |  |
| 400 | 78 | F | 13.32 |  |  |
| 401 | 95 | M |  |  | 12.33 |
| 402 | 68 | M |  | 11.52 |  |
| 403 | 88 | M |  |  | 13.69 |
| 404 | 83 | F | 15.14 |  |  |
| 405 | 90 | F |  | 11.46 |  |
| 406 | 73 | F |  |  | 9.14 |
| 407 | 70 | M | 11.5 |  |  |
| 408 | 92 | M |  |  | 12.05 |
| 409 | 75 | M |  |  | 11.57 |
| 410 | 83 | F |  |  | 11.98 |
| 411 | 88 | M |  | 10.86 |  |
| 412 | 85 | M |  |  | 9.25 |
| 413 | 85 | F |  |  | 12.83 |
| 414 | 73 | F | 12.46 |  |  |
| 415 | 73 | F | 12.91 |  |  |
| 416 | 73 | M |  |  | 12.12 |
| 417 | 71 | F | 11 |  |  |
| 418 | 88 | M | 12 |  |  |
| 419 | 71 | F | 12.32 |  |  |
| 420 | 68 | M | 9.89 |  |  |
| 421 | 58 | M |  | 14.81 |  |
| 422 | 81 | M |  |  | 12.04 |
| 423 | 71 | M | 13.27 |  |  |
| 424 | 65 | F | 13.36 |  |  |
| 425 | 85 | F |  |  | 11.41 |
| 426 | 67 | M | 12.05 |  |  |
| 427 | 67 | F |  |  | 13.17 |
| 428 | 91 | F |  | 10.75 |  |
| 429 | 72 | M |  | 11.09 |  |
| 430 | 80 | M |  |  | 11.75 |
| 431 | 72 | M | 12.44 |  |  |
| 432 | 68 | M | 12.54 |  |  |
| 433 | 88 | M |  |  | 11.97 |
| 434 | 93 | F |  |  | 13.39 |
| 435 | 98 | F |  |  | 12.79 |
| 436 | 85 | M |  |  | 14.56 |
| 437 | 86 | F | 14.4 |  |  |
| 438 | 85 | M |  | 12.6 |  |
| 439 | 78 | M |  |  | 11.47 |
| 440 | 52 | M |  | 12.82 |  |
| 441 | 77 | M |  | 13.06 |  |
| 442 | 67 | M | 9.22 |  |  |
| 443 | 78 | F | 11.54 |  |  |
| 444 | 79 | M | 15.28 |  |  |
| 445 | 82 | M |  |  | 9.59 |
| 446 | 88 | F |  |  | 11.49 |
| 447 | 72 | M |  |  | 12.43 |
| 448 | 85 | M |  |  | 12.83 |
| 449 | 67 | M |  |  | 14.25 |
| 450 | 79 | M |  |  | 12.47 |
| 451 | 79 | M |  | 13.23 |  |
| 452 | 80 | F |  | 12.03 |  |
| 453 | 66 | M |  |  | 13.38 |
| 454 | 100 | M |  |  | 9.42 |
| 455 | 98 | M | 9.33 |  |  |
| 456 | 83 | F |  |  | 9.15 |
| 457 | 70 | F |  |  | 9.31 |
| 458 | 89 | M | 8.4 |  |  |
| 459 | 86 | M |  | 7.82 |  |
| 460 | 70 | F |  | 7.91 |  |
| 461 | 75 | F | 11.78 |  |  |
| 462 | 65 | F | 7.58 |  |  |
| 463 | 86 | M |  |  | 10.29 |
| 464 | 75 | M |  |  | 7.68 |
| 465 | 66 | M |  |  | 16.06 |
| 466 | 72 | F |  |  | 11.46 |
| 467 | 86 | F |  |  | 11.29 |
| 468 | 82 | M |  |  | 9.41 |
| 469 | 86 | M |  | 9.96 |  |
| 470 | 85 |  | 8.8 |  |  |
| 471 | 81 | F | 9.82 |  |  |
| 472 | 83 | F | 12.81 |  |  |
| 473 | 82 | F | 8.44 |  |  |
| 474 | 85 | M |  |  | 10.91 |
| 475 | 60 | M |  |  | 11.26 |
| 476 | 68 | F |  | 11.06 |  |
| 477 | 80 | F |  | 10.39 |  |
| 478 | 82 | F |  | 11.85 |  |
| 479 | 73 | M | 10.63 |  |  |
| 480 | 90 | F |  | 9.66 |  |
| 481 | 75 | F |  | 10.48 |  |
| 482 | 70 | M |  |  | 9.73 |
| 483 | 80 | M |  | 9.08 |  |
| 484 | 60 | M |  | 12.42 |  |
| 485 | 87 | F | 12.22 |  |  |
| 486 | 87 | F |  | 11.8 |  |
| 487 | 73 | M | 10.56 |  |  |
| 488 | 77 | M | 9.08 |  |  |
| 489 | 81 | M |  |  | 11.37 |
| 490 | 77 | M | 11.18 |  |  |
| 491 | 82 | F |  |  | 13.13 |
| 492 | 75 | M |  | 11.7 |  |
| 493 | 72 | M | 9.67 |  |  |
| 494 | 65 | M |  | 12.75 |  |
| 495 | 71 | F | 14.39 |  |  |
| 496 | 70 | F |  |  | 11.44 |
| 497 | 91 | M |  |  | 13.11 |
| 498 | 71 | F |  |  | 11.66 |
| 499 | 80 | F |  |  | 15.14 |
| 500 | 71 | F |  | 14.46 |  |
| 501 | 66 | M |  | 13.37 |  |
| 502 | 86 | M |  | 10.91 |  |
| 503 | 62 | M | 9.87 |  |  |
| 504 | 86 | F |  | 11.13 |  |
| 505 | 71 | F |  | 15.12 |  |
| 506 | 81 | M |  |  | 12.97 |
| 507 | 71 | M |  |  | 10.25 |
| 508 | 87 | M |  |  | 11.04 |
| 509 | 75 | F |  |  | 12.25 |
| 510 | 5 | F |  |  | 13.11 |
| 511 | 30 | M | 9.82 |  |  |
| 512 | 0 | F |  |  | 11.87 |
| 513 | 0 | F |  |  | 13.77 |
| 514 | 0 | F |  |  | 13.05 |
| 515 | 1 | F |  |  | 11.23 |
| 516 | 50 | M |  |  | 12.59 |
| 517 | 4 | F |  |  | 12.2 |
| 518 | 59 | M |  |  | 12.31 |
| 519 | 33 | M | 13.54 |  |  |
| 520 | 51 | F |  |  | 12.63 |
| 521 | 0 | F |  |  | 14.8 |
| 522 | 0 | M |  |  | 11.43 |
| 523 | 0 | M |  |  | 16.31 |
| 524 | 18 | M |  |  | 13.35 |
| 525 | 0 | M |  |  | 17.51 |
| 526 | 42 | F |  |  | 15.88 |
| 527 | 53 | F | 13.6 |  |  |
| 528 | 0 | M |  |  | 13.18 |
| 529 | 0 | M |  |  | 15.73 |
| 530 | 58 | M | 12.42 |  |  |
